# Assessing *Wolbachia* circulation in wild populations of phlebotomine sand flies from Spain and Morocco: implications for control of leishmaniasis

**DOI:** 10.1186/s13071-025-06771-6

**Published:** 2025-04-26

**Authors:** Andrés Torres-Llamas, Victoriano Díaz-Sáez, Manuel Morales-Yuste, Patricia Ibáñez-De Haro, Arturo Enrique López-López, Victoriano Corpas-López, Francisco Morillas-Márquez, Joaquina Martín-Sánchez

**Affiliations:** Department of Parasitology, Faculty of Pharmacy, Campus Universitario de Cartuja, University of Granada, 18071 Granada, Spain

**Keywords:** *Leishmania*, *Phlebotomus perniciosus*, *Phlebotomus sergenti*, *Wolbachia* prevalence, Haplotype, WSP, Biological control

## Abstract

**Background:**

Vector-borne diseases such as leishmaniasis exert a huge burden of morbidity and mortality that are mainly controlled through vector control. The increasing threat of insecticide-resistant vectors entails incorporating more vector control interventions to eliminate these diseases. Introduction of *Wolbachia* into wild vector populations has been suggested as a potential vector control measure that would require extensive regional knowledge. The aim of this work is to estimate the prevalence of *Wolbachia* infection and monitor circulating strains in wild sand fly populations from Spain and Morocco, two countries where leishmaniasis is endemic.

**Methods:**

*Wolbachia* was detected using polymerase chain reaction (PCR). Haplotype diversity was performed by sequencing, and phylogenetic relationships were then established. In silico prediction of the *Wolbachia* surface protein (WSP) structures was performed. To investigate the relationship between epidemiological variables and the presence of *Wolbachia*, regression analyses were employed.

**Results:**

*Wolbachia* was detected in 45.8% of the specimens tested (319/697), and similar infection rates were found (*P* = 0.92) in males (46.1%; 94/204) and females (45.6%; 225/493). Differences in infection were detected among Spanish sand fly species (*P* < 0.001), being higher for *Phlebotomus papatasi* (35/52) and *Phlebotomus perniciosus* (239/384). No infected *Phlebotomus sergenti* specimens were found in Spain, whereas two different *Wolbachia* haplotypes were detected in *P. sergenti* sand flies from Morocco. No significant differences were found between sex, species, or capture sites in specimens captured in Morocco (*P* > 0.05). Five *Wolbachia* haplotypes distributed in the known A and B supergroups were identified. Structural analysis showed a nine-amino acid insertion in the fourth loop of a *Wolbachia* haplotype found in *P. sergenti* specimens from El Borouj (Morocco).

**Conclusions:**

We confirmed the circulation of different *Wolbachia* strains in all sand fly species investigated. All *L. infantum* proven or suspected vectors shared the same, or a closely related, *Wolbachia* haplotype. The haplotype bearing the loop insertion was found in the locality undergoing an anthroponotic cutaneous leishmaniasis outbreak. These extracellular loops might have some role in enhancing or inhibiting the development of *Leishmania* and other pathogens in sand flies. These findings are very promising and highlight the need to further investigate the tripartite interactions between *Wolbachia* strain, *Leishmania* species, and sand fly species/lineage.

**Graphical Abstract:**

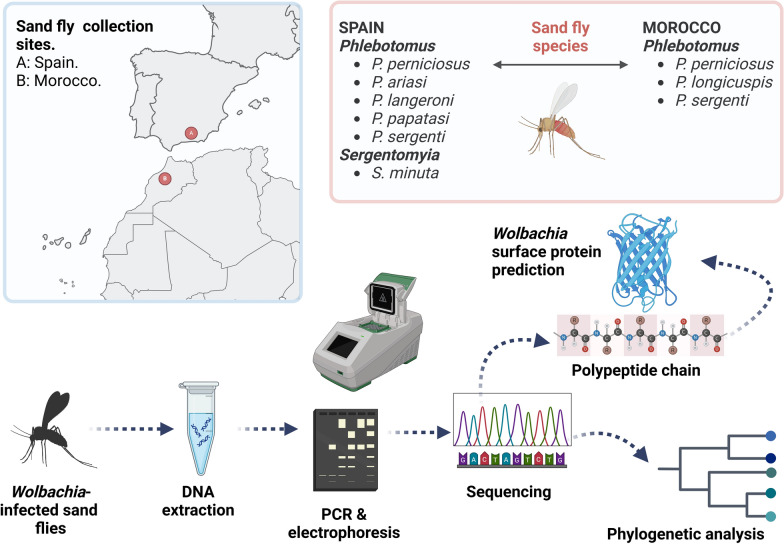

**Supplementary Information:**

The online version contains supplementary material available at 10.1186/s13071-025-06771-6.

## Background

Leishmaniasis is a vector-borne disease caused by protozoan parasites of the genus *Leishmania* and transmitted by the bite of infected female sand flies (Diptera, Psychodidae, Phlebotominae). Approximately 1 million new infections occur each year showing two main clinical forms, visceral leishmaniasis (VL), which is fatal if untreated, and cutaneous leishmaniasis (CL) [1]. *Leishmania donovani* and *Leishmania infantum* are the main causative agents of VL, among the 20 *Leishmania* species involved in the clinical spectrum of leishmaniasis. CL is a disfiguring and stigmatizing parasitic disease that is caused by a variety of *Leishmania* species [1]. Morocco is considered as 1 of the 12 “high-burden countries” for CL, where it is caused mainly by two species, *Leishmania major* and *Leishmania tropica,* while VL is caused by *L. infantum* [2]*. L. tropica* has the widest geographic distribution in this country and has shown a highly emerging nature, being able to expand rapidly into nonendemic areas [2]. *L. infantum* is the only species identified as a causative agent of both CL and VL diseases [3]. Furthermore*, L. infantum* is the most widely distributed species, being the only one present both in the New World and in the Old World [1]. As in most European countries of the Western Mediterranean Basin, human leishmaniasis is hypoendemic in Spain. However, VL and Cl are underreported and underdiagnosed, particularly CL [4, 5], while there is no anthroponotic CL (ACL) due to *L. tropica* despite the existence of the vector, *Phlebotomus sergenti* [6].

Six species of sand flies (*Phlebotomus perniciosus*, *Phlebotomus papatasi*, *Phlebotomus ariasi*, *Phlebotomus sergenti*, *Phlebotomus langeroni*, and *Sergentomyia minuta*) are commonly found in sympatry in the biotopes studied in southern Spain. Three of them, all from *Larroussius* subgenus, have been implicated in the transmission of *L. infantum* in southwestern Europe: *P. perniciosus*, which is the main vector, *P. ariasi*, and *P. langeroni* [7]. *Phlebotomus longicuspis* is a *Larroussius* species that is only present in North Africa where it is generally found in sympatry with *P. perniciosus*, and the limitations of morphology to differentiate these species have been highlighted [8].

Vector-borne diseases are mainly prevented through vector control strategies among which biological control emerges as a promising alternative to classic chemical control with insecticides. *Wolbachia pipientis* is an intracellular obligate bacterium (Alphaproteobacteria, Rickettsiales, Anaplasmataceae) transmitted maternally, which infects most insect species, including sand flies [9, 10]. *Wolbachia* infection influences insect development by causing reproductive alterations such as parthenogenesis, feminization, male killing, and cytoplasmic incompatibility (CI) [10, 11]. In the latter, embryonic death occurs when infected males mate with an uninfected female (CI unidirectional) or females infected with an incompatible *Wolbachia* strain (CI bidirectional) [12, 13].

*Wolbachia* has been detected in several sand fly species from both the New and the Old World [14–16]. In addition, different species of sand flies have been found to be infected by the same *Wolbachia* strain, suggesting a phenomenon of horizontal cross-species transmission [17]. The CI phenotype and the success of vertical transmission to future generations have been assessed in laboratory-reared *P. papatasi* from Egypt, showing that both CI and maternal transmission partially occur in these insects [18, 19]. These facts support the possibility of using this endosymbiont as a method of biological control of *Leishmania* vectors. Studies of the prevalence of the endosymbiont under natural conditions are required, as well as studies to identify the infecting *Wolbachia* strains in sand flies from various areas. Environmental factors such as temperature extremes appear to have a negative impact on *Wolbachia* density, thus compromising the *Wolbachia* prevalence in a host population. However, susceptibility to temperature depends mostly on the genetic conditions of the host itself and the symbionts [20, 21]. All of this will influence *Wolbachia* circulation in wild populations of vectors.

The primary objective of this study was to estimate the prevalence of *Wolbachia* infection and monitor circulating *Wolbachia* strains in wild sand fly populations from Spain and Morocco. Our focus has been particularly on *P. perniciosus* and *P. sergenti*, the principal vectors of zoonotic leishmaniasis caused by *L. infantum* and ACL due to *L. tropica*, respectively. Ultimately, we aim to determine the *Wolbachia* haplotype diversity through *wsp* gene analysis and predict structural variations in its surface protein via amino acid analysis, which may provide insights into the mechanisms underlying host interactions.

## Methods

### Study area

The study was conducted in Granada Province (southern Spain) and Settat Province (central Morocco). In Granada Province, a continental climate predominates in most of the region, while a mountain climate is evident at higher altitudes (reaching up to 3479 m above sea level in Sierra Nevada). In addition, a subtropical climate prevails along the coast. The principal economic activities are agriculture and tourism. In contrast, Settat Province encompasses a vast expanse of predominantly rural territory characterized by an arid to semi-arid temperate and cold climate. The region’s economy primarily hinges on agriculture and livestock farming.

### Sand fly collection and species identification

**Fig. 1:**
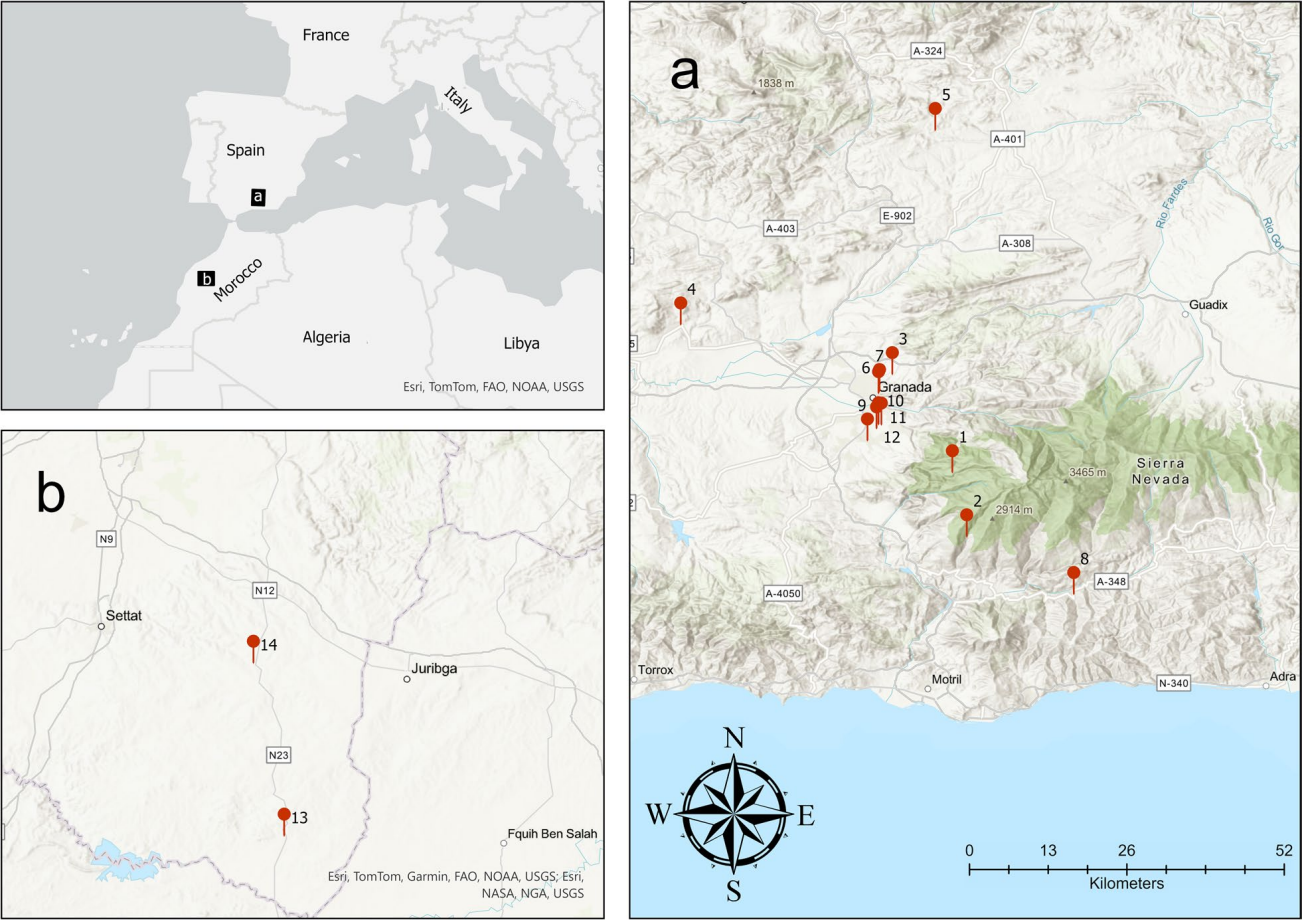
Geographic distribution of sand fly collection sites in Spain (**a**) and Morocco (**b**). 1, Collado; 2, Ballesteros; 3, Mantas; 4, Íllora; 5, Montejícar; 6, Albaicín; 7, Sacromonte; 8, Torvizcón; 9, Gójar; 10, Zubia 1; 11, Zubia 2; 12, Zubia 3; 13, El Borouj; 14, Sidi Hajjaj

Phlebotomine sand flies were collected using Centers for Disease Control and Prevention (CDC) light traps at 14 locations (Table 1, Fig. 1). Specimens from Spain were collected from 2019 to 2023 and those from Morocco were collected in 2015. All specimens were collected from 20 June to 10 July and from 20 September to 10 October. The Moroccan specimens were morphologically identified and processed for DNA extraction in previous studies [8, 22]. Spanish males and females were morphologically identified using the taxonomic keys, particularly considering the spermatheca in females and the external genitalia in males [7, 23, 24]. Moroccan and Spanish specimens were processed under identical conditions. The determination of *P. longicuspis *sensu stricto was carried out by sequencing and phylogenetic analyses of different mitochondrial and nuclear genes, as shown in a previous study [8]. In addition, 27 Spanish *P. sergenti* specimens caught elsewhere in Spain were tested for *Wolbachia* infection [6, 25].

**Table 1:** Geographical locations where sand flies were captured and molecularly analyzed

Province, country	Location	Altitude (m.a.s.l)	Coordinates	No. of specimens
Granada, Spain	Collado	1813	37°03′50.5"N 3°28′48.7"W	44 (44 F)
Granada, Spain	Ballesteros	1753	36°58′08.1"N 3°27′31.9"W	20 (20 F)
Granada, Spain	Mantas	885	37°12′34.5"N 3°34′9.5"W	288 (79 M + 209 F)
Granada, Spain	Íllora	759	37°16′59.9"N 3°52′59.9"W	54 (22 M + 32 F)
Granada, Spain	Montejícar	1137	37°34′19.0"N 3°30′20.0"W	2 (2 F)
Granada, Spain	Albahicín	780	37°10′51.0"N 3°35′23.0"W	1(1 F)
Granada, Spain	Sacromonte	860	37°11′04"N 3°35′18" W	11(11 F)
Granada, Spain	Torvizcón	685	36°53′ 00"N 3°18′ 00"W	9 (5 M + 4 F)
Granada, Spain	Gójar	760	37°6′40.0"N 3°36′22.3"W	8 (8 F)
Granada, Spain	Zubia 1	700	37°8′6.8"N 3°35′23.0"W	13 (13 F)
Granada, Spain	Zubia 2	740	37°8′5.1"N 3°35′8.10"W	1(1 F)
Granada, Spain	Zubia 3	695	37°7′44.9"N 3°35′33.6"W	133 (67 M + 66 F)
Settat, Morocco	El Borouj	410	32°30′47″N 7°11′44″W	86 (29 M + 57 F)
Settat, Morocco	Sidi Hajjaj	547	33°06′43"N 07°24′21"W	27 (2 M + 25 F)
Total	–	–	–	697 (204 M + 493 F)

M, male; m.a.s.l, meters above sea level; F female

### Sand fly DNA extraction

Genomic DNA was extracted from head, thorax, and attached anterior abdomen of individual male and female sand flies. A commercially MasterPure^™^ Complete DNA and RNA Purification Kit (cat. no. MC85200) was used according to the manufacturer´s instructions. Each sand fly was individually placed in a sterile 1.5 mL Eppendorf tube and kept in liquid nitrogen for a few seconds to facilitate the mechanic rupture of the tissues using a pestle. The DNA was resuspended in 20 µL of bidistilled water and kept at −20 °C until use. DNA concentration and purity were analyzed using Thermo Scientific NanoDrop^™^ 2000c spectrophotometer.

### Detection of *Wolbachia* DNA

The presence of *Wolbachia* DNA in sand flies was investigated using a conventional PCR technique. A set of primers described by Zhou et al. [26] were used to amplify a fragment of 591–632 base pairs (bp) of the *Wolbachia wsp* gene: 81F (5′ -TGG TCC AAT AAG TGA TGA AGA AAC—3′) and 691R: (5′- AAA AAT TAA ACG CTA CTC CA −3′). These primers have allowed the detection of various *Wolbachia* strains in sand flies from both the New World and the Old World [16, 26, 27]. PCR was performed in 25 µL of reaction volume. Briefly, 16 µL of bidistilled water, 2.5 µL 10X buffer, 0.5 µL 50 mM MgCl_2_, 1 µL 10 mM dNTP_s,_ 0.5 µL 25 mM forward and reverse primers, 1 µL 5 U/µL Taq polymerase, and 3 µL sand fly DNA were added to the reaction tube. The amplification conditions were: 2 min denaturation at 94 °C followed by 35 cycles of denaturation at 94 °C for 30 s, annealing at 55 °C for 30 s, and extension at 72 °C for 1 min, with a final extension at 72 °C for 10 min. PCR products were separated by electrophoresis in a 1.5% agarose gel, and the size was determined by comparison with HyperLadder 100 bp (Bioline, Meridian Bioscience, UK).

### Sequencing and phylogenetic analysis

Amplified PCR products were eluted from agarose gel using Real Clean Spin Kit (Zymoclean^™^ Gel DNA Recovery Kit, cat. no. D4007), according to the manufacturer’s protocol. Direct cycle sequencing of the PCR product was performed in both directions by an automated sequencer using the same primers used for DNA amplification. Sequences obtained were visualized and manually adjusted with Chromas version 2.6.6 and then aligned using the multiple sequence alignment program Clustal Omega (https://www.ebi.ac.uk/Tools/msa/clustalo/). Polymorphic sites as well as DNA polymorphism were analyzed with DNAsp version 6.12.03. The selection of the best nucleotide substitution model was carried out with the jModeltest version 2.1.10 software and MEGA11. Analysis was performed using MEGA11 to infer phylogenetic relationships of *Wolbachia wsp* consensus sequences detected in sand flies and others available in GenBank. The genetic distances were calculated using the Kimura 2-parameter model (K2P). Three different methods were used: maximum likelihood (ML), neighbor-joining (NJ), and maximum parsimony (MP). NJ tree was inferred by using the p-distance model. ML tree was inferred by using the Tamura 3-parameter substitution model with gamma distribution (T92 + G), which showed Akaike information criterion (AIC) score of 4814.086. MP tree was inferred by using max-mini branch-and-bound search method. All positions that contained gaps and missing data were removed from the analyses (complete deletion). Robustness of the internal branches was evaluated by bootstrap analysis through 1000 bootstrap replications, and the values below 50% were hidden. *Bemisia tabaci* (FJ404651) sequence was used as outgroup.

### In silico *Wolbachia* surface protein (WSP) structure prediction and confidence analysis

**Fig. 2:**
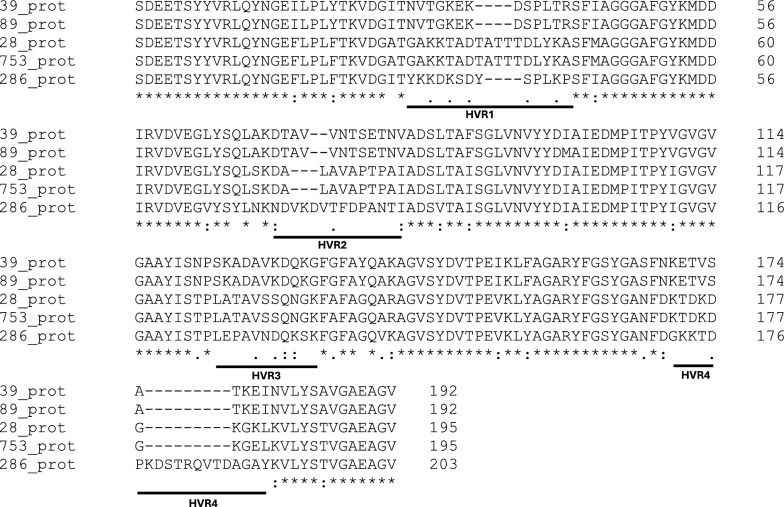
Amino acid sequence alignment of the five *Wolbachia* haplotypes detected in this study. The four regions of hypervariability that coincide with the four WSP loops are shown. (*), conserved sites; (:), sites with conservative replacements; (.), sites with semi-conservative replacements; blank space, sites with non-conservative replacements

All nucleotide sequences were translated into amino acid sequences and then aligned (Fig. 2). Three-dimensional structures of the peptides were predicted using ColabFold version 1.5.3., which is based on Alphafold2 and MMseq2 [28]. Using ColabFold’s server, five structures were generated for each peptide with sub-models of AlphaFold. Structural templates from PDB100 were used as input along with the peptide sequences for better accuracy. The predicted structures were relaxed using the Amber force field, which helps remove stereochemical violations in predictions [29, 30]. The five structures for each sequence were ranked by the per-residue estimate, using the predicted local-distance difference test (pLDDT) as AlphaFold’s confidence measure—a metric used by AlphaFold to estimate local confidence at the level of individual amino acid residues in a predicted protein structure: regions with very high confidence (pLDDT > 90), confidently predicted regions (90 > pLDDT > 70), regions of low confidence (70 > pLDDT > 50), and very low confidence regions (pLDDT < 50). Likewise, the predicted template modeling score (pTM) was used to assess the overall quality of the structure prediction of the complete protein; a score close to 1 means that the overall fold predicted for the protein will be similar to the true structure [29, 31]. Finally, the Protein Data Bank (PDB) file of the Amber relaxed rank 1 structure for each peptide (Additional File 1: Supplementary Dataset S1) was visualized with Biovia Discovery Studio 2021 software (Dassault Systèmes BIOVIA, San Diego, CA, USA) for structural analysis. The approximate size of the protein was made with Avogadro version 1.2.0.

### Data analysis

Sand fly capture locations were georeferenced on the basis of their longitude and latitude (Table 1, Fig. 1). To investigate a possible association between environmental temperature and *Wolbachia* infection in sand flies, both logistic regression and linear regression analyses were performed, depending on whether the dependent variable was temperature or the presence of *Wolbachia*. For this purpose, monthly maximum, minimum, and average temperature data available on WorldClim 2.1 (https://www.worldclim.org/), at a spatial resolution of 2.5 min and consisting of Geotiff files, were obtained. Subsequently, a “shapefile” with the different temperature layers corresponding to each month of the year for each sampling location was created and then opened using ArcGis Pro version 3.1 (ESRI). Finally, the temperature values were extracted and exported in xlsx format.

Other variables, such as sex and species of phlebotomine sand flies, altitude, sampling site, and country, were analyzed to find an association with the presence of *Wolbachia*. These characteristics were used as independent variables, while the presence/absence of *Wolbachia* in sand fly specimens were set as the dependent variable. Logistic regression was the statistical method of choice.

All tests were conducted using IBM SPSS^®^ Statistics version 21.0 for Windows (IBM Corp., Armonk, NY, USA). A two-step procedure was used to select the most parsimonious model. First, all explanatory variables were assessed in a univariate model; variables significant at the *P* ≤ 0.2 level were included in the final multivariate model (both, logistic, or linear). Then, we used a stepwise backward elimination procedure. Variables with a* P*-value greater than 0.05 were removed. The potential for confusion and interaction between the independent variables was controlled.

## Results

### *Wolbachia* infection: prevalence and factors associated with the presence of endosymbiont

The presence of *Wolbachia* was assessed in 697 sand fly specimens captured in 14 sampling locations (Table 1). There were 204 males (29.3%) and 493 females (70.7%) belonging to seven species and two genera (*Phlebotomus* and *Sergentomyia*). In Spain, 584 specimens were collected: 173 males (29.7%) and 410 females (70.3%); the species identified were *P. perniciosus* (65.9%, *n* = 385); *P. sergenti* (9.2%, *n* = 54); *P. papatasi* (8.9%, *n* = 52); *Sergentomyia minuta* (7.9%, *n* = 46); *P. langeroni* (4.3%, *n* = 25), and *P. ariasi* (3.8%, *n* = 22). Table 2 lists these specimens together with the analyzed specimens from Morocco.

**Table 2:** *Wolbachia* infection in phlebotomine sand fly species from different geographical locations

	*P. perniciosus*		*P. sergenti*		*P. papatasi*		*S. minuta*		*P. longicuspis*		*P. langeroni*		*P. ariasi*		Total	
	M	F	M	F	M	F	M	F	M	F	M	F	M	F	M	F
1	–	26 (11)	–	–	–	–	–	–	–	–	–	–	–	18 (1)	–	44 (12)
2	–	19 (13)	–	–	–	–	–	–	–	–	–	–	–	1 (0)	–	20 (13)
3	72 (46)	181 (117)	3 (0)	2 (0)	4 (2)	4 (1)	–	22 (11)	–	–	–	–	–	–	79 (48)	209 (129)
4	14 (4)	15 (9)	–	–	–	–	–	–	–	–	8 (0)	17 (2)	–	–	22 (4)	32 (11)
5	–	–	–	–	–	–	–	–	–	–	–	–	–	2 (0)	–	2 (0)
6	–	–	–	–	–	–	–	–	–	–	–	–	–	1 (0)	–	1 (0)
7	–	–	–	11 (0)	–	–	–	–	–	–	–	–	–	–	–	11 (0)
8	–	–	5 (0)	4 (0)	–	–	–	–	–	–	–	–	–	–	5 (0)	4 (0)
9	–	8 (5)	–	–	–	–	–	–	–	–	–	–	–	–	–	8 (5)
10	–	8 (2)	–	–	–	–	–	5 (0)	–	–	–	–	–	–	–	13 (2)
11	–	1 (0)	–	–	–	–	–	–	–	–	–	–	–	–	–	1 (0)
12	22 (17)	19 (15)	12 (0)	17 (0)	26 (20)	18 (12)	7 (0)	12 (1)	–	–	–	–	–	–	67 (37)	66 (28)
13	7 (0)	11 (2)	10 (1)	20 (6)	–	–	–	–	12 (3)	26 (10)	–	–	–	–	29 (4)	57 (18)
14	–	–	2 (1)	25 (7)	–	–	–	–	–	–	–	–	–	–	2 (1)	25 (7)
Total	115 (67)	288 (174)	32 (2)	79 (13)	30 (22)	22 (13)	7 (0)	39 (12)	12 (3)	26 (10)	8 (0)	17 (2)	–	22 (1)	204 (94)	493 (225)

1, Collado; 2, Ballesteros; 3, Mantas; 4, Íllora; 5, Montejícar; 6, Albahicín; 7, Sacromonte; 8, Torvizcón; 9, Gójar; 10, Zubia 1; 11, Zubia 2; 12, Zubia 3; 13, El Borouj; 14, Sidi Hajjaj. M, male; F, female; number in brackets indicates the number of sand flies positive for *Wolbachia*

*Wolbachia wsp* was successfully amplified in 45.8% of the specimens tested (319/697). Globally, *Wolbachia* infection rate was similar in male (46.1%; 94/204) and female (45.6%; 225/493) sand flies (*P* = 0.92), and also when only the species *P. perniciosus* was considered (*P* = 0.69).

*Wolbachia* was found in 289/584 sand fly specimens captured in Spain (50.4%), and this infection rate was similar (*P* = 0.557) in males (89/173, 51.4%) and females (200/411, 48.7%). Differences in *Wolbachia* infection rate were detected among Spanish sand fly species (*P* < 0.001). Infection rate was found to be higher in *P. perniciosus* (239/384) than *P. ariasi* (1/22; *P* = 0.001, odds ratio (OR) = 0.029; 95% confidence interval (CI) 0.004–0.217), *P. langeroni* (2/25; *P* < 0.001, OR = 0.053; 95% CI 0.012–0.227), and *S. minuta* (12/46; *P* < 0.001, OR = 0.214; 95% CI 0.107–0.427), whereas *P. papatasi* had a similar infection rate (35/52; *P* = 0.478). *Wolbachia* was not found in *P. sergenti* (0/54). Differences in *Wolbachia* infection rates were also detected among the Spanish sand fly capture sites, both when all sand fly species were considered (*P* < 0.001), and when only *P. perniciosus* (*P* = 0.01) was considered. The presence of *Wolbachia* in the highest altitude sampling site (Collado, Table 1) was significantly lower (*P* < 0.001, OR = 0.392; 95% CI 0.186–0.827) than in other sites (Table 2). However, no association was found between *Wolbachia* infection and the altitude as a continuous variable (*P* = 0.399). No association was found between *Wolbachia* infection in *P. perniciosus* and any of the temperature variables, either by linear (dependent variable = temperature) or logistic regression (dependent variable = *Wolbachia* presence/absence); (*P* > 0.5).

*Wolbachia* was found in 30/113 sand fly specimens captured in Morocco (26.5%), and the infection rate was not significantly different (*P* = 0.130) between males (5/31, 16.1%) and females (25/82, 30.5%). No difference in *Wolbachia* infection was found either among sand fly species (*P* = 0.96) or among sampling sites (*P* = 0.68). When only the species *P. sergenti* was considered, no difference was detected in *Wolbachia* infection associated with the sand fly sex (*P* = 0.40) or the capture area (*P* = 0.59).

The presence of *Wolbachia* was higher among *P. perniciosus* specimens captured in Spain than in Morocco (*P* < 0.001), being 13-fold higher for Spanish *P. perniciosus* specimens than for *P. perniciosus* in El Borouj (OR = 13.10, 95% CI 2.97–57.80). In contrast, no infection was found in *P. sergenti* specimens captured in Spain, whereas in Morocco *Wolbachia* infection was found in specimens from both localities surveyed, El Borouj and Sidi Hajjaj.

### Characterization of *Wolbachia* haplotypes

Five haplotypes of the *Wolbachia wsp* gene were identified among the DNA sequences of all sand fly species analyzed, all found in Spain and Morocco (haplotype diversity = 1.0; nucleotide diversity (Pi) = 0.14700). Sequence alignment of the 621-bp fragment showed 143 polymorphic sites and 55 indels (insertion/deletion sites) at DNA level (31.9% mutation rate). A total of 115 parsimony-informative sites were also found among the *wsp* sequences in the different sand fly species. In Spain, *Wolbachia* haplotype detected in *P. perniciosus*, *P. ariasi*, *P. langeroni*, and *S. minuta* corresponded to *Wolbachia* strain wPrn isolated in *P. perniciosus* from Italy (AF237884), with 100% homology. However, the *Wolbachia* haplotype detected in *P. papatasi* was 100% identical to *Wolbachia* wPap of *P. papatasi* detected in different countries, such as India (AF237882) and Iran (MG251659).

In Morocco, the *Wolbachia* haplotype found in *P. perniciosus* was the same as that detected in Spain. Nevertheless, the *Wolbachia* haplotype found in *P. longicuspis* s.s. had a single nucleotide polymorphism (SNP) compared with that of *P. perniciosus* at position 297, generating a change in the amino acid sequence (isoleucine to methionine) at residue 99, between hypervariable regions 2 and 3 (Fig. 2). Two *Wolbachia* haplotypes were detected in *P. sergenti* specimens from Morocco. *Wolbachia* haplotype detected in *P. sergenti* from Sidi Hajjaj had only a SNP compared with that of *P. papatasi* at position 541; the SNP generated an amino acid change (glutamic acid to lysine) at residue 181, in the fourth hypervariable region (Fig. 2). The *Wolbachia* haplotype found in *P. sergenti* from El Borouj showed a higher relationship with haplotypes of the endosymbiont detected in species of the genus *Drosophila* with 98.8–99.8% nucleotide sequence homology.

Between the two *wsp* haplotypes found in *P. sergenti*, each in a Moroccan locality, 75 polymorphic sites and 48 indels were found. Overall, the genetic variation at amino acid level among the *Wolbachia* sequences of this study was 38%. Of this percentage, 20.2% were indels and 79.8% were substitutions. Analysis in DNAsp highlighted that most mutations were non-synonymous, with the percentage of amino acid variation (38%) being higher than the mutation rate at the DNA level (31.9%). The genetic distance values among the *Wolbachia* sequences obtained in this study ranged between 0.000 and 0.215.

### Phylogenetic analysis of the *Wolbachia* of sand flies

**Fig. 3:**
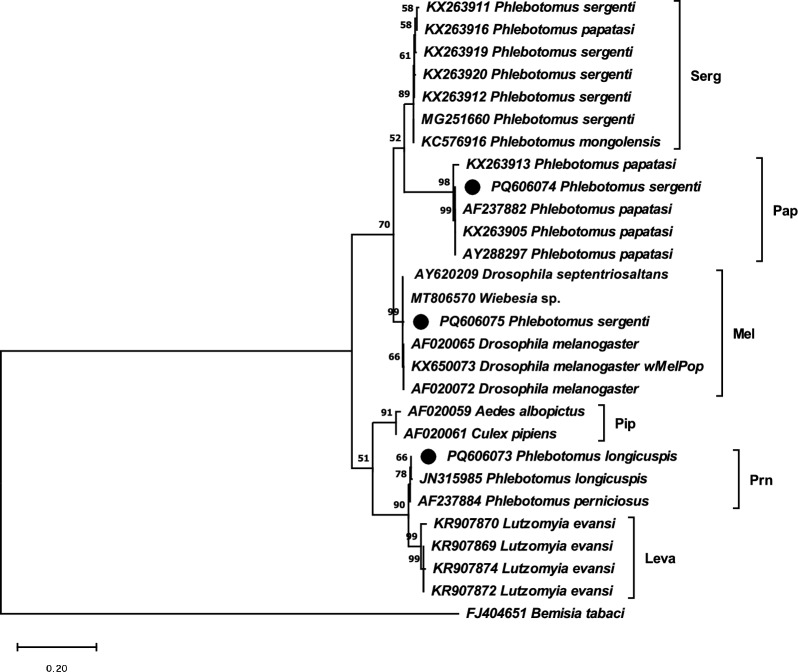
Maximum likelihood tree drawn to scale showing the phylogenetic relationships and distribution into the different subgroups of the *wsp* haplotypes detected in this study (marked with a circle) and others from GenBank

In total, 27 *Wolbachia* sequences detected in sand flies and other taxa belonging to *Drosophila*, *Aedes*, and *Culex* genera available in GenBank were used to build a phylogenetic tree. Among the sequences used, five are derived from the present study, with three newly sequenced entries now deposited in GenBank under the following accessions: PQ606073–PQ606075. The three methods used to infer phylogenetic relationships (NJ, ML, and MP) showed a similar topology. Maximum likelihood tree drawn to scale is shown (Fig. 3). All sequences were grouped into two clearly distinct clusters (A and B supergroups, bootstrap values > 50%). Furthermore, six subgroups of *Wolbachia* were differentiated and supported by the bootstrap values: Serg, Mel, and Pap (A supergroup) and Pip, Prn, and Leva (B supergroup). The sequence of *Wolbachia* detected in *P. perniciosus*, *P. ariasi*, *P. langeroni*, and *S. minuta* clustered into the Prn subgroup, such as the one detected in *P. longicuspis* s.s., whereas that detected in *P. papatasi* clustered into the Pap subgroup. *Wolbachia* haplotypes detected in *P. sergenti* from Morocco clustered either within the Pap (Sidi Hajjaj) or within Mel subgroups (El Borouj, Fig. 3).

### Three-dimensional WSP structure prediction based on *Wolbachia* amino acid sequences

**Fig. 4:**
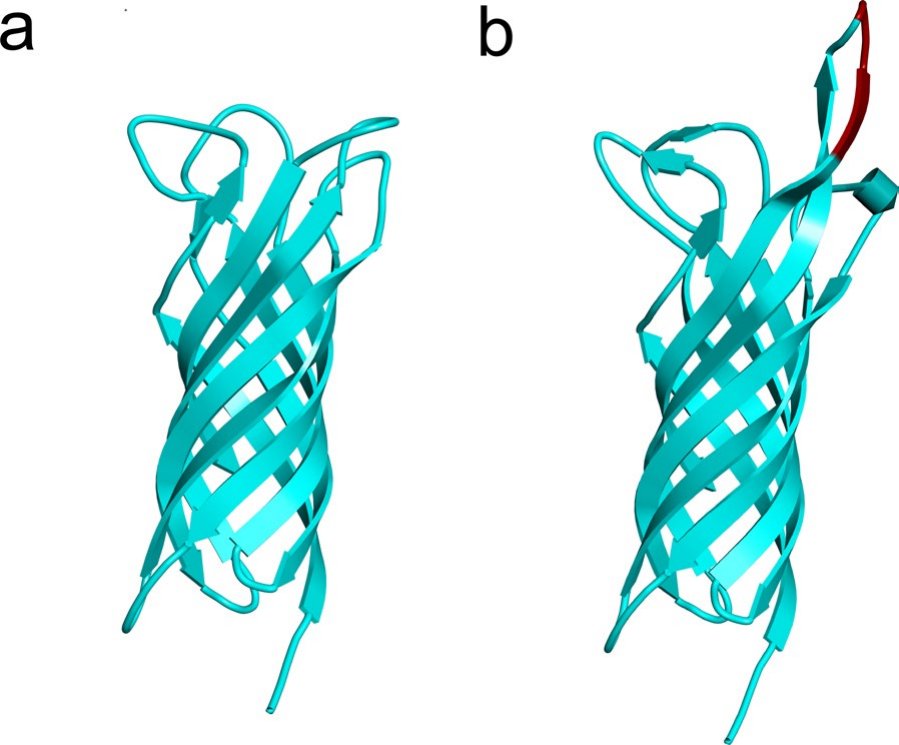
Prediction of WSP structures using Colabfold software. The WSP haplotype found *P. perniciosus* from Spain, and El Borouj (**a**) is shown in comparison with another WSP detected in *P. sergenti* from El Borouj (**b**). The insertion of nine amino acids that causes the elongation of the fourth loop is marked in red (**b**)

All the sequences analyzed contain a typical beta-barrel structure consisting of eight antiparallel β-sheets, with an approximate size of 60 × 25 Å. pLDDT and pTM scores above 90 and 0.85, respectively, were obtained, resulting in predicted structural models with high reliability. The proteins have four mostly hydrophilic loops with a poorly defined structure. The regions of major divergence in the amino acid sequence known as hypervariability regions (HVR1, HVR2, HVR3, and HVR4) coincide with the loops located on the extracellular face. The polymorphism detected between the two wPrn haplotypes found in this study results in an equivalent amino acid change (conservative), i.e., isoleucine to methionine (Fig. 2). In contrast, the amino acid change detected between both wPap haplotypes (glutamic acid to lysine), one from *P. papatasi* (Spain) and another from *P. sergenti* from Sidi Hajjaj (Morocco), respectively, is non-conservative (Fig. 2). Regarding the *Wolbachia* strain found in *P. sergenti* from El Borouj, the presence of a nine-amino acid fragment (KDSTRQVTD) that is absent in the rest of the haplotypes stands out. The fragment is found in the fourth loop, causing an elongation of approximately 10 Å in the shape of a beta-sheet (Fig. 4).

## Discussion

Vector-borne diseases exert a huge burden of morbidity and mortality worldwide, particularly affecting the poorest populations. With the rising threat from insecticide-resistant vectors, there is a need to incorporate more vector interventions to control these diseases such as bacterial infection of vectors [32]. In this regard, there is increased interest in exploiting *Wolbachia* as a means of biological control of arthropod transmitted infectious pathogens on the basis of its ability to significantly alter the biology of the host in the symbiotic relationship, mainly host reproduction through CI. Therefore, it is important to carry out further studies to know the extent of *Wolbachia* infections in field-collected sand flies and perform their characterization to detect the existence of different strains.

In the present study, we have estimated the prevalence of infection by sex and species and identified different *Wolbachia* haplotypes in wild populations of sand flies from two countries where leishmaniasis is endemic, Spain and Morocco. We detected *Wolbachia* infection in all species investigated and in both sexes. Previous studies of *Wolbachia* infection in sand flies from both the Old World and the New World have shown a disparity of results in the relative proportions of infection in both sexes and a possible higher infection rate in females than males [15, 27, 33, 34]. The infection rates by species vary from 80.7% to 4.5%, with maximum values for *P. papatasi* and minimum values for *P. ariasi.* The two species with the highest prevalence values were *P. perniciosus* and *P. papatasi*, with figures to these found by other authors elsewhere [15, 17]. This is the first time that *P. ariasi* and *P. langeroni* have been found naturally infected by this endosymbiont, despite low infection rates.

*Wolbachia* was not detected in any individual of *P. sergenti* from Spain; to confirm this finding, other specimens (*n* = 27) captured elsewhere in Spain [6, 25] were tested, confirming the absence of *Wolbachia* infection in this sand fly species. However, these results should be taken with caution owing to the size of the sample analyzed (*n* = 81). In contrast, high *Wolbachia* infection rates have been found in *P. sergenti* in the two Moroccan localities included in this study and in Iranian endemic foci of ACL [17]. The *Wolbachia* load and, consequently, the prevalence of infection in a host population appear to be influenced by ecological and environmental factors, the host itself, and the *Wolbachia* strain [20, 35–37].

The existence of a predominant *P. sergenti* mitochondrial lineage has been demonstrated in Spain [25], as well as in each of the two Moroccan localities included in this study [22], being different from each other. It has been suggested that the main factor that would explain the absence of ACL in an area could be the existence of a determined *P. sergenti* mitochondrial lineage. The genetic background of the host may also help to understand the differences found in *Wolbachia* prevalence in *P. perniciosus* from Spain versus Morocco. The specimens of this sand fly species belong to two different mitochondrial (mt) DNA sublineages. The Spanish *P. perniciosus* belong to the Iberian sublineage and the Moroccan ones to the typical mt DNA sublineages [8].

Concerning the environmental factors, the results of the studies are contradictory, and it is unclear whether an increase in temperature enhances or reduces bacterial density [21, 38]. In this study, we did not find any association of environmental temperature or altitude (both variables being continuous) with the *Wolbachia* infection. It is likely that our sample does not include enough sampling sites spanning a wide range of altitudes, each with an adequate number of specimens. However, we found significant differences between *Wolbachia* infection in sand flies captured at different sampling sites in Spain, among which there are altitudinal differences. The highest presence of the endosymbiont was detected at 885 m.a.s.l. and the lowest at 1813 m.a.s.l., the highest altitude sampled. Since altitudinal changes appear to be negatively correlated with temperature [39], these results might support the idea expressed above that it is extreme temperatures, in this case the coldest ones, that lead to a reduction in *Wolbachia* presence, although this needs to be further investigated.

All *Larroussius* species in Spain, *P. perniciosus*, *P. ariasi*, and *P. langeroni*, proven vectors of *L. infantum*, share the same *Wolbachia* haplotype that matches the wPrn strain found in Italy and France [40, 41]. *Phlebotomus perniciosus* specimens from Morocco also share the genotype wPrn. Although *Wolbachia* has previously been found in *P. longicuspis* s.s. from Morocco [42], we have detected a novel *Wolbachia* haplotype that differs from the previous one in a single nucleotide at position 196. These haplotypes are very close to wPrn, so when the three sequences were compared, only two polymorphic sites were found. A haplotype close to wPrn was also detected in another *Larroussius* sand fly, *P. perfiliewi*, different only by a single ambiguous base at nucleotide 409 [16, 34].

*Wolbachia* was previously described in *S. minuta* from France and was found to share high homology (99%) with the endosymbiont of *Tribolium confusum* [41]. Nevertheless, in this study we detected the presence of the wPrn strain in all infected individuals of *S. minuta*; there are wide genetic differences between both haplotypes (29 polymorphic sites). Despite being considered a herpetophilous species, increasing records of blood meals in mammals, including humans, and the frequent detection of *L. infantum* DNA suggested that it could be involved in the transmission of *L. infantum*. However, other authors have suggested that the low parasitic loads found in *S. minuta* do not allow for the transmission of the parasite [43, 44].

While the presence of the endosymbiont was not detected in the Spanish *P. sergenti* specimens, the Moroccan specimens from two nearby localities with radical epidemiological differences showed similar infection rates due to two different *Wolbachia* haplotypes. In Sidi Hajjaj, a leishmaniasis nonendemic area, we found the wPap strain, whereas the wMel haplotype (98.8–99.8% homology) was found in El Borouj, an area undergoing an ACL outbreak [2, 22]. In addition, 13 *Wolbachia wsp* sequences detected in *P. sergenti* were available in GenBank, and they showed wide differences with the genotypes identified in the present study (322–363 polymorphisms and 59–99 indels). Differences in both the density and the mitochondrial lineage among *P. sergenti* populations were detected between these two Moroccan localities that could explain the different epidemiological trend [22], but the existence of different genotypes of the *Wolbachia* endosymbiont may as well be relevant.

The finding that different species found in sympatry share the same *Wolbachia* strain has been previously described [17, 34, 41, 45]. Occupying the same ecological niche can enhance interactions between species, whether phylogenetically close or not. Therefore, such host–host interactions could result in sharing microbiome symbionts, as *Wolbachia* in sand flies [46]. Horizontal transmission of *Wolbachia* has been shown plausible through different mechanisms, including predation [47], interactions through parasitoids/parasites [48], or sharing food sources [49].

However, to explain the diversity of *Wolbachia* strains generally detected (five different haplotypes were found in our study), some authors have suggested the existence of introgression between strains of the endosymbiont after hybridization of closely related host species [50, 51]. There is evidence for introgression of both nuclear and mitochondrial DNA between some phlebotomine sand fly species from both the New World and the Old World [52, 53]. Such host hybridizations could be accompanied by the interaction of their microbiomes, including intracellular endosymbionts.

In recent years, the importance of the outer membrane proteins of symbiont bacteria in the invasion and proliferation of bacteria in vertebrate and invertebrate hosts has been highlighted [54]. *Wolbachia* surface protein (WSP) is a transmembrane protein located in the outermost membrane of the endosymbiont, which has a beta-barrel structure, and eight antiparallel sheets connected to four extracellular loops, which constitute the protein’s major areas of variability [55, 56]. Several studies have shown that WSP is able to stimulate the immune response of humans infected with some filarial species, either by activation of the inflammatory response through Toll-like receptor (TLR-2 and TLR-4) pathways [57] or inhibition of apoptosis of cells of the polymorphonuclear system [58]. More recently, it has been shown that TEP1 and APL1 genes, which are related to *Plasmodium* sp. death, were overexpressed in *Anopheles gambiae* cells when challenged with recombinant WSP, compared with cells derived from *Aedes albopictus*, a natural *Wolbachia* host [59].

There is scarce information on how *Wolbachia* interacts with sand flies and *Leishmania*. Varoto-Bocazzi et al. [60] showed that WSP from a *Wolbachia* strain isolated from *Dirofilaria immitis* is able to activate the classical macrophage response causing the death of *Leishmania* amastigotes in vitro. Likewise, Da silva and colleagues tested the introduction of wMel and wMelPop-CLA strains into two cell lines from *Lutzomyia longipalpis* and challenged them against a *L. infantum* strain. They concluded that bacterial proliferation depends on the strain and cell line in question. However, they found that wMelPop-CLA activates the main immunity pathways, but there was no significant interaction with *L. infantum* [61].

*Wolbachia*–*Leishmania* interactions remain to be elucidated, as most co-infection studies have been carried out in the New World, and there is a variety of results ranging from protection to high rates of co-infection [45, 62–66]. Extracellular loops of WSP appear to be responsible for stimulating the immune response by inducing the release of several cytokines as well as by inducing the expression of molecules such as adhesins that allow bacteria to bind to host cells [67]. In addition, they are also thought to be involved in the recognition of exogenous ligands [68]. Our results revealed a similar structure of all the WSP haplotypes found, except for the haplotype found in *P. sergenti* from El Borouj, which presents a nine-amino acid insertion in the fourth loop that is absent in the other haplotypes. The largest areas of variation at the amino acid level were found in the four extracellular loops. All these polymorphisms are thought to be the result of mutation or rather recombination that these *Wolbachia* proteins have undergone as a means of evolutionary adaptation [55, 69].

These polymorphisms found in the loops (including the nine-amino acid insertion) of WSP could be involved in epitope recognition of host immune system molecules related to bacterial survival and proliferation [69, 70]. We hypothesize that there may be a relationship between the *Wolbachia* strain and the sand fly species/lineage, which may somehow enhance or inhibit the development of *Leishmania*. We believe that future research should focus on the study of interactions between *Wolbachia* strains (based on their WSP) with the sand fly host as well as with the protozoan *Leishmania* spp. in both in vitro and in vivo models. Prior to this, further epidemiological studies are required to identify the circulating *Wolbachia* strains in nature and what impact they might have on the invertebrate host and subsequently *Leishmania* development.

## Conclusions

We confirmed the circulation of different *Wolbachia* strains in all the sand fly species investigated and in both sexes. All *L. infantum* proven or suspected vectors shared the same, or very related, *Wolbachia* haplotype. Regarding the species *P. sergenti*, while the presence of the endosymbiont was not detected in the Spanish specimens, the Moroccan specimens from two nearby localities with radical epidemiological differences showed similar infection rates due to two different *Wolbachia* haplotypes that showed structural differences in the fourth loop of the WSP. These extracellular loops could have some role in enhancing or inhibiting the development of *Leishmania* and other pathogens in sand flies. These findings are very promising and highlight the need to further investigate the tripartite interactions between *Wolbachia* strain, *Leishmania* species, and sand fly species/lineage.

## Supplementary Information

Additional File 1: Dataset S1. Structural predictions of WSP for each of the *Wolbachia* haplotypes found in the sand flies in this study. Prediction confidence values are shown. The input amino acid sequence as well as templates used in modelling from PDB100 are shown.

Additional File 2: Dataset S2.

Additional File 3: Dataset S3.

Additional File 4: Dataset S4.

Additional File 5: Dataset S5.

## Data Availability

Data is provided within the manuscript or supplementary information files. Three sequences have been deposited in GenBank under the following accessions: PQ606073–PQ606075.

## Abbreviations

CL: Cutaneous leishmaniasis
VL: Visceral leishmaniasis
ACL: Anthroponotic cutaneous leishmaniasis
CI: Cytoplasmic incompatibility
s.s.: Sensu stricto
WSP: *Wolbachia* surface protein
HVR: Hypervariability region
