# Supplementary figures and images for: Assessing Wolbachia circulation in wild populations of phlebotomine sand flies from Spain and Morocco: implications for control of leishmaniasis
Source: Parasit Vectors. 2025 Apr 26;18:155. doi: 10.1186/s13071-025-06771-6 (PMC12032678; doi:10.1186/s13071-025-06771-6)

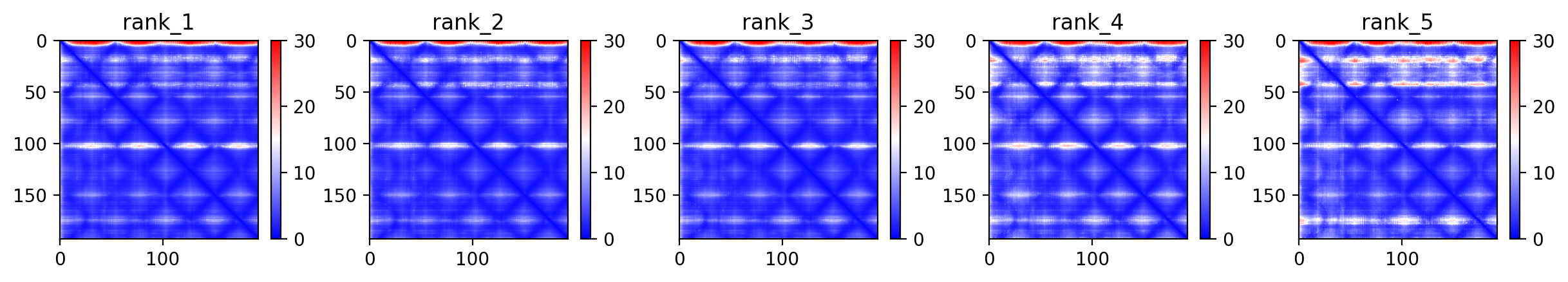

Supplement: Supplementary file 1 — Additional File 1: Dataset S1. Structural predictions of WSP for each of the Wolbachia haplotypes found in the sand flies in this study. Prediction confidence values are shown. The input amino acid sequence as well as templates used in modelling from PDB100 are shown. [file 13071_2025_6771_MOESM1_ESM.zip › Phlebotomuslongicuspis_02e8e/Phlebotomuslongicuspis_02e8e_pae.png]

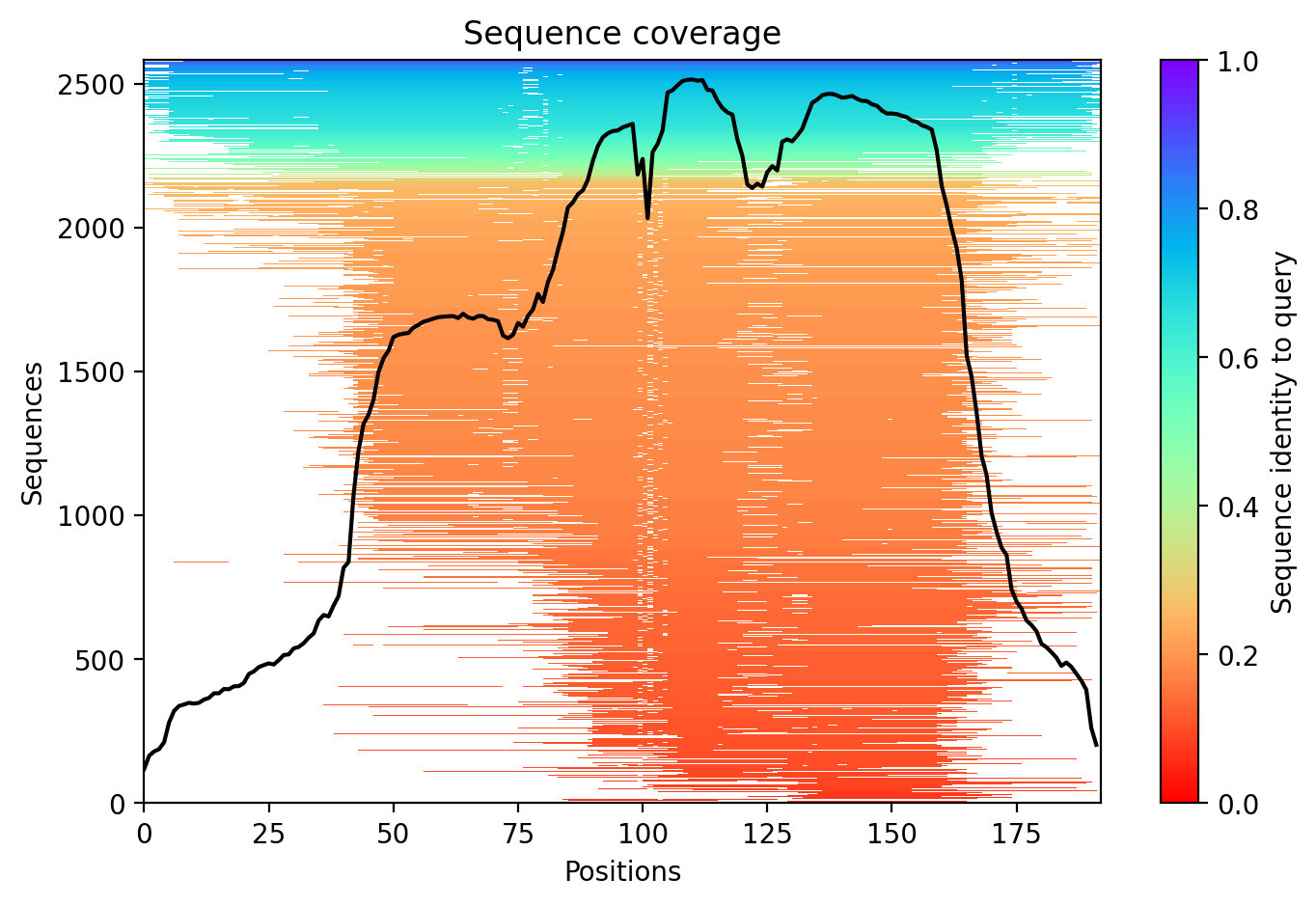

Supplement: Supplementary file 1 — Additional File 1: Dataset S1. Structural predictions of WSP for each of the Wolbachia haplotypes found in the sand flies in this study. Prediction confidence values are shown. The input amino acid sequence as well as templates used in modelling from PDB100 are shown. [file 13071_2025_6771_MOESM1_ESM.zip › Phlebotomuslongicuspis_02e8e/Phlebotomuslongicuspis_02e8e_coverage.png]

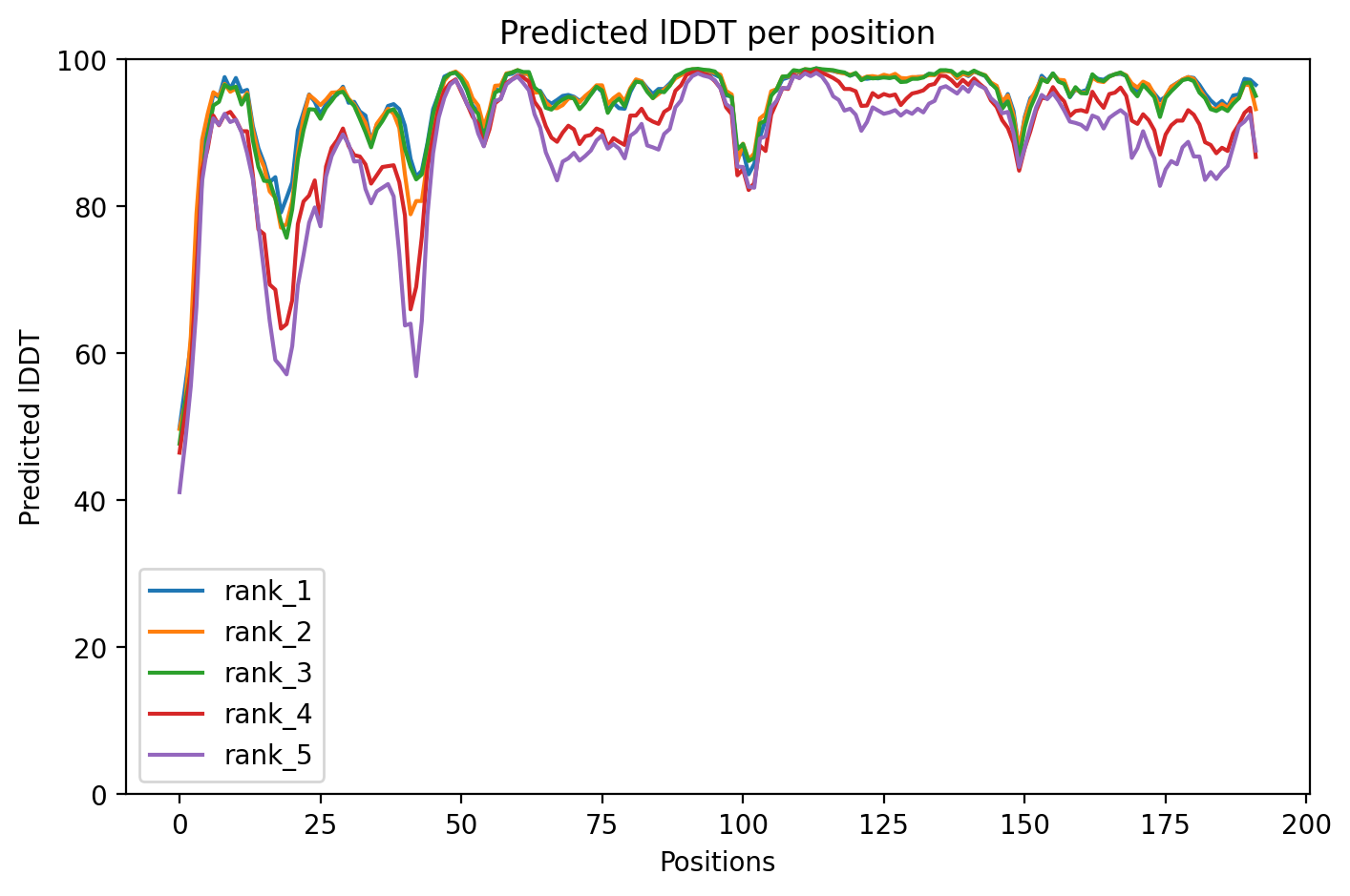

Supplement: Supplementary file 1 — Additional File 1: Dataset S1. Structural predictions of WSP for each of the Wolbachia haplotypes found in the sand flies in this study. Prediction confidence values are shown. The input amino acid sequence as well as templates used in modelling from PDB100 are shown. [file 13071_2025_6771_MOESM1_ESM.zip › Phlebotomuslongicuspis_02e8e/Phlebotomuslongicuspis_02e8e_plddt.png]

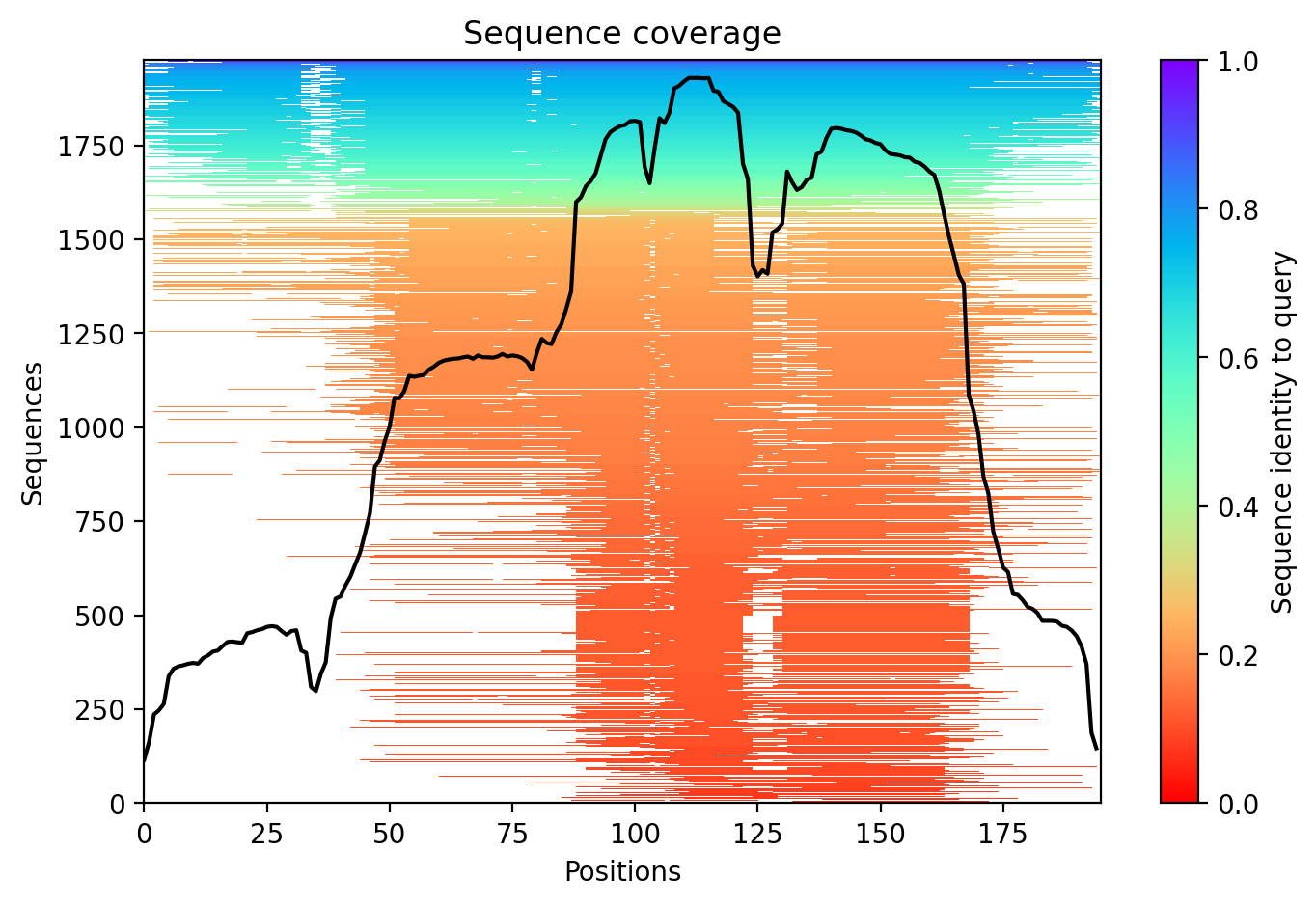

Supplement: Supplementary file 2 — Additional File 2: Dataset S2. [file 13071_2025_6771_MOESM2_ESM.zip › Phlebotomuspapatasi_6ff74/Phlebotomuspapatasi_6ff74_coverage.png]

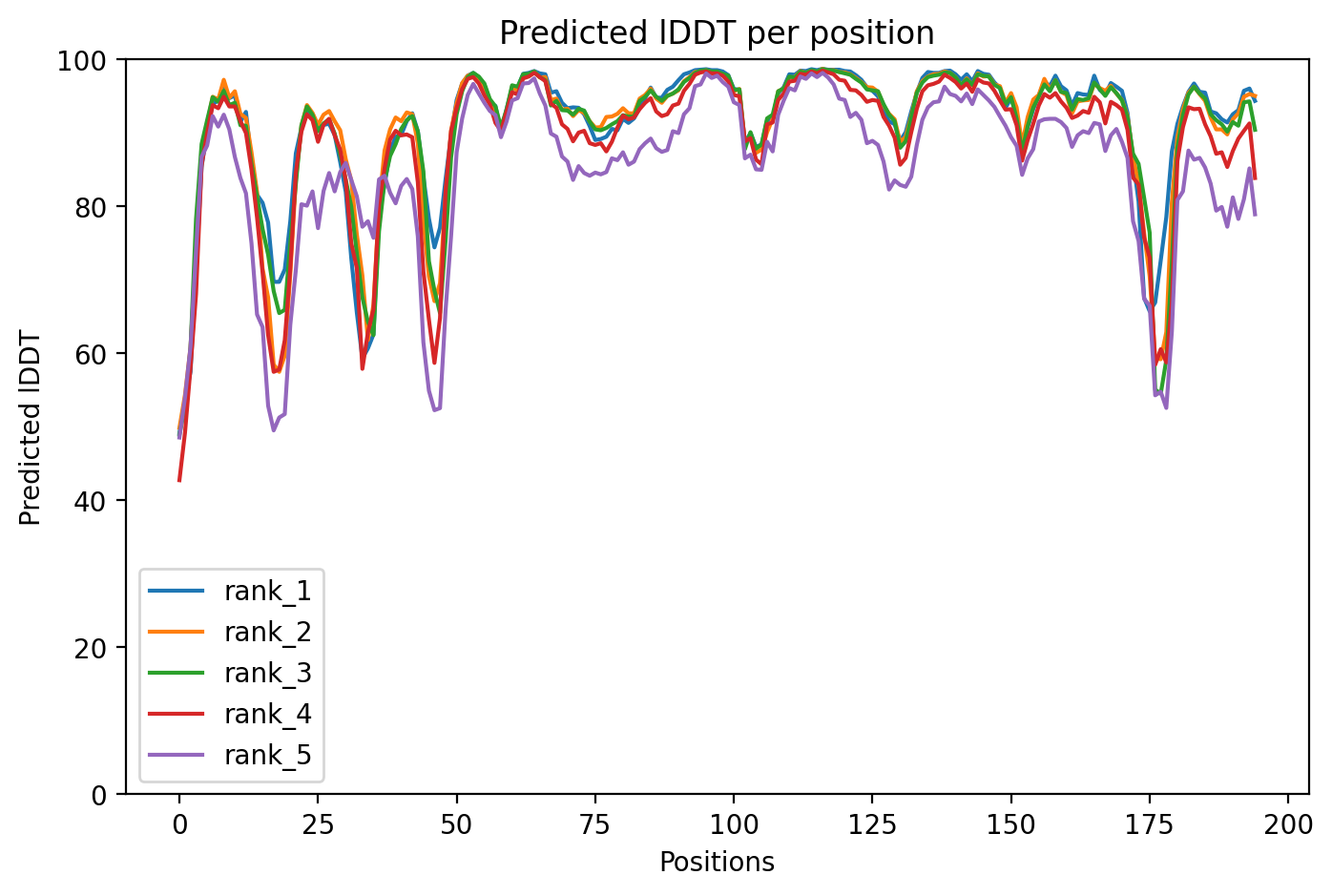

Supplement: Supplementary file 2 — Additional File 2: Dataset S2. [file 13071_2025_6771_MOESM2_ESM.zip › Phlebotomuspapatasi_6ff74/Phlebotomuspapatasi_6ff74_plddt.png]

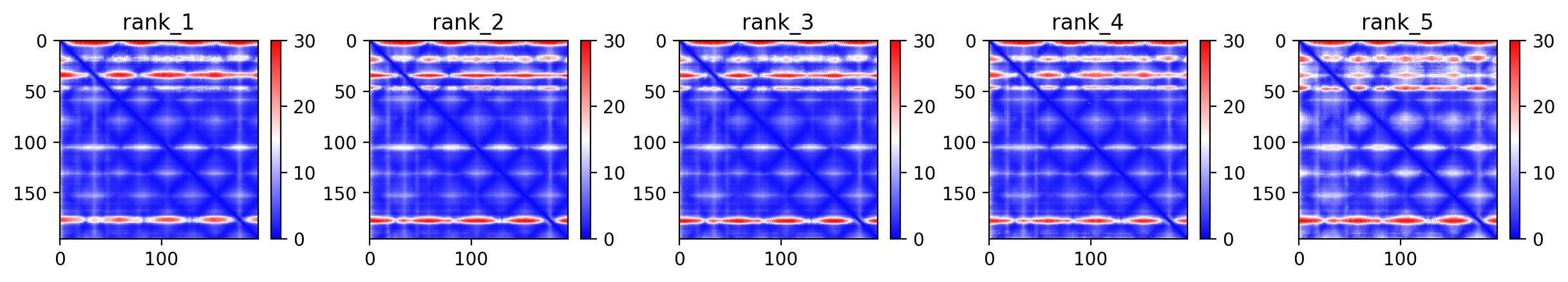

Supplement: Supplementary file 2 — Additional File 2: Dataset S2. [file 13071_2025_6771_MOESM2_ESM.zip › Phlebotomuspapatasi_6ff74/Phlebotomuspapatasi_6ff74_pae.png]

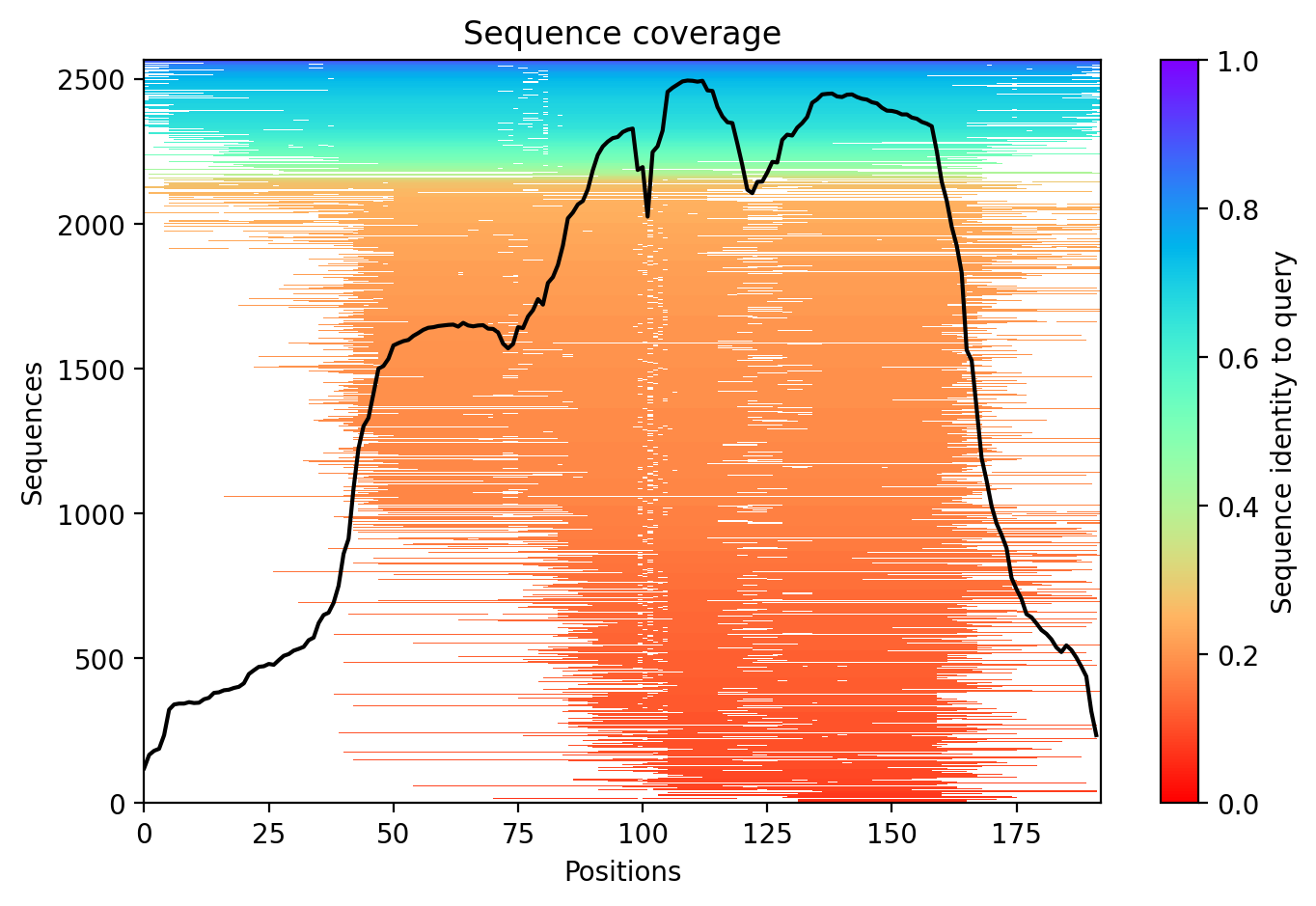

Supplement: Supplementary file 3 — Additional File 3: Dataset S3. [file 13071_2025_6771_MOESM3_ESM.zip › Phlebotomusperniciosus_bfd4b/Phlebotomusperniciosus_bfd4b_coverage.png]

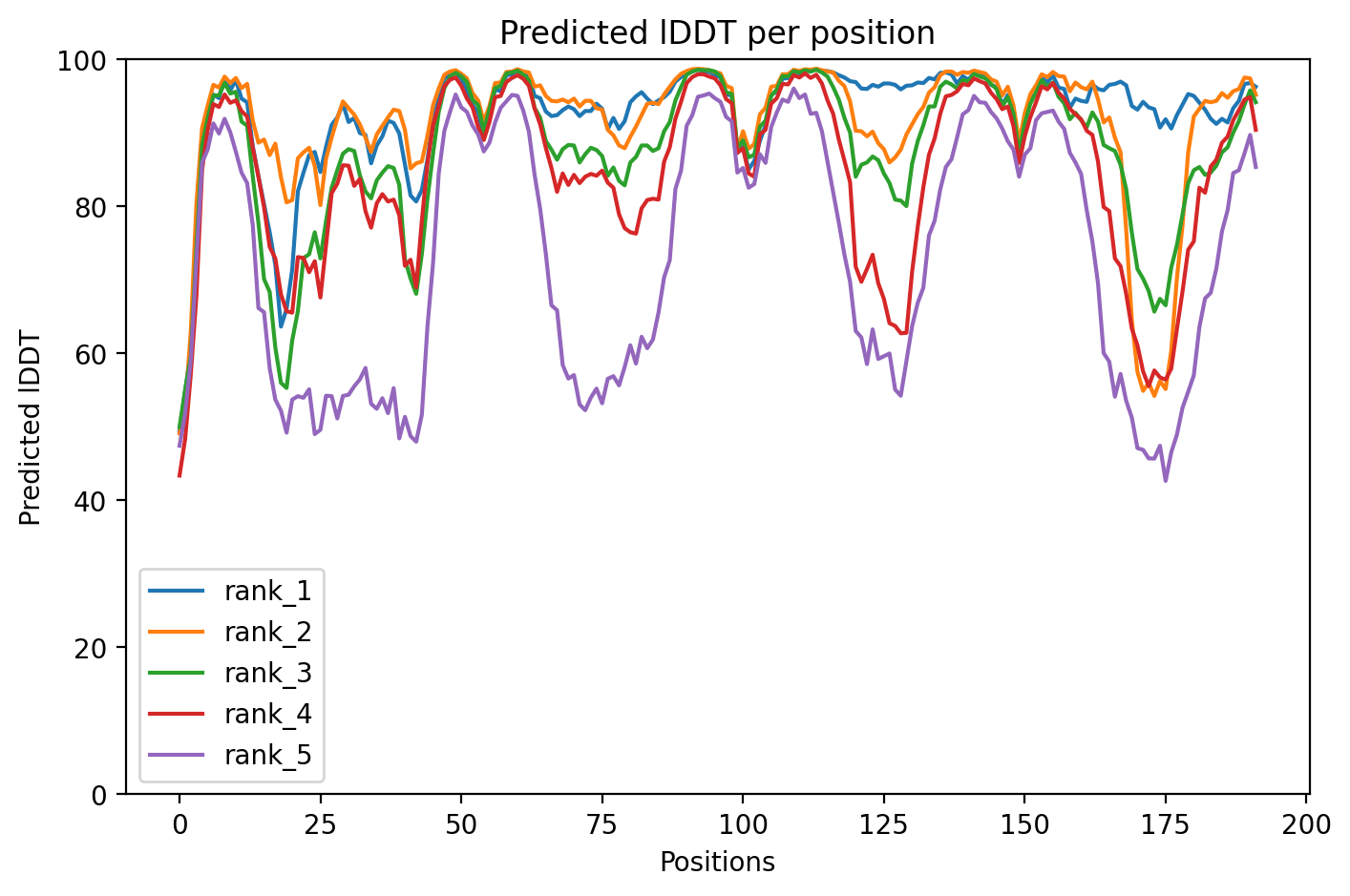

Supplement: Supplementary file 3 — Additional File 3: Dataset S3. [file 13071_2025_6771_MOESM3_ESM.zip › Phlebotomusperniciosus_bfd4b/Phlebotomusperniciosus_bfd4b_plddt.png]

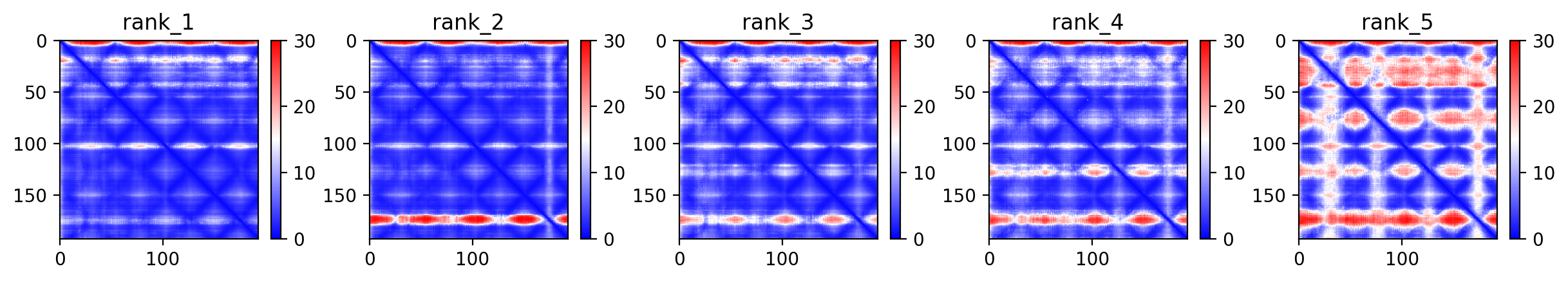

Supplement: Supplementary file 3 — Additional File 3: Dataset S3. [file 13071_2025_6771_MOESM3_ESM.zip › Phlebotomusperniciosus_bfd4b/Phlebotomusperniciosus_bfd4b_pae.png]

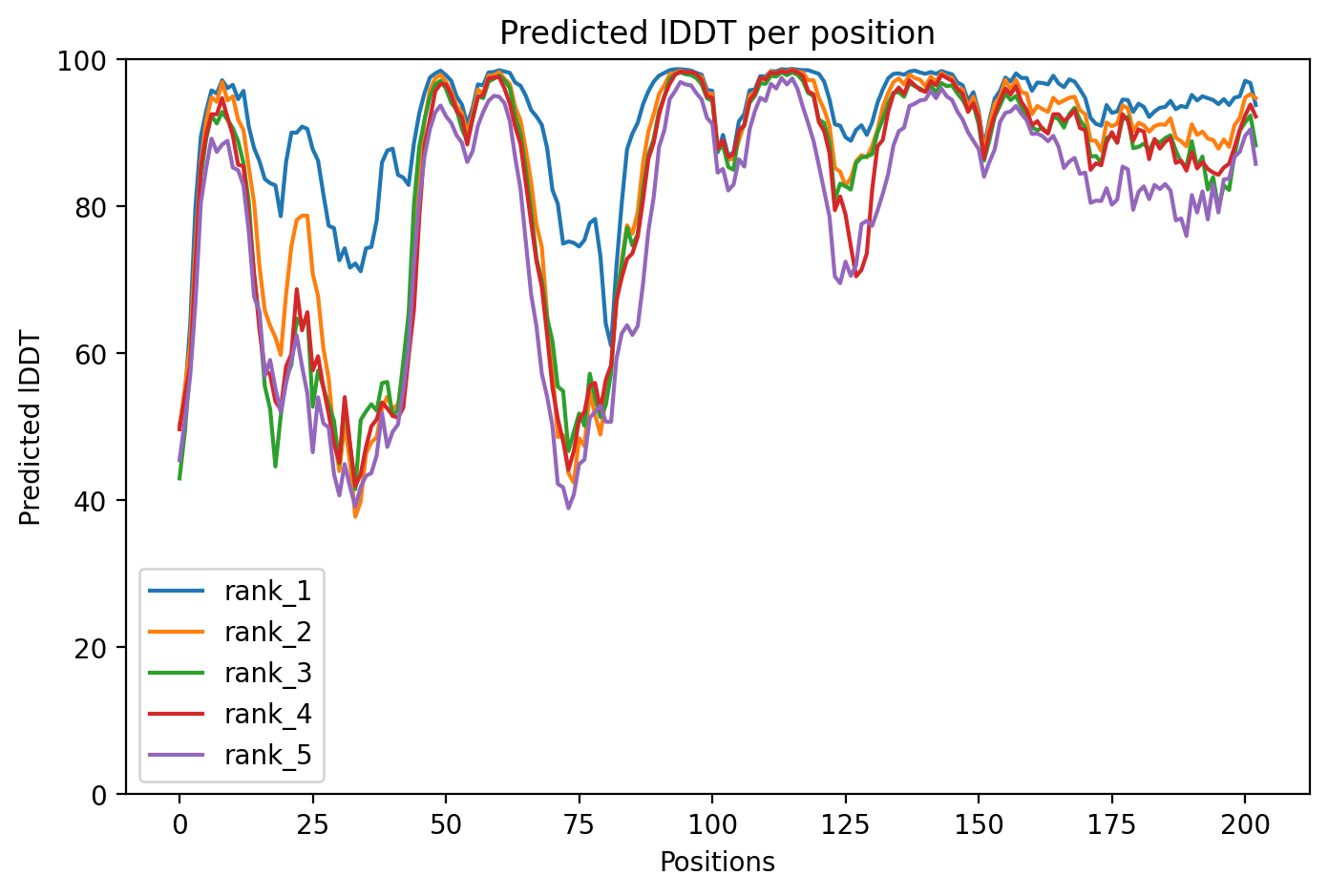

Supplement: Supplementary file 4 — Additional File 4: Dataset S4. [file 13071_2025_6771_MOESM4_ESM.zip › PhlebotomussergentiEB_01ca0/PhlebotomussergentiEB_01ca0_plddt.png]

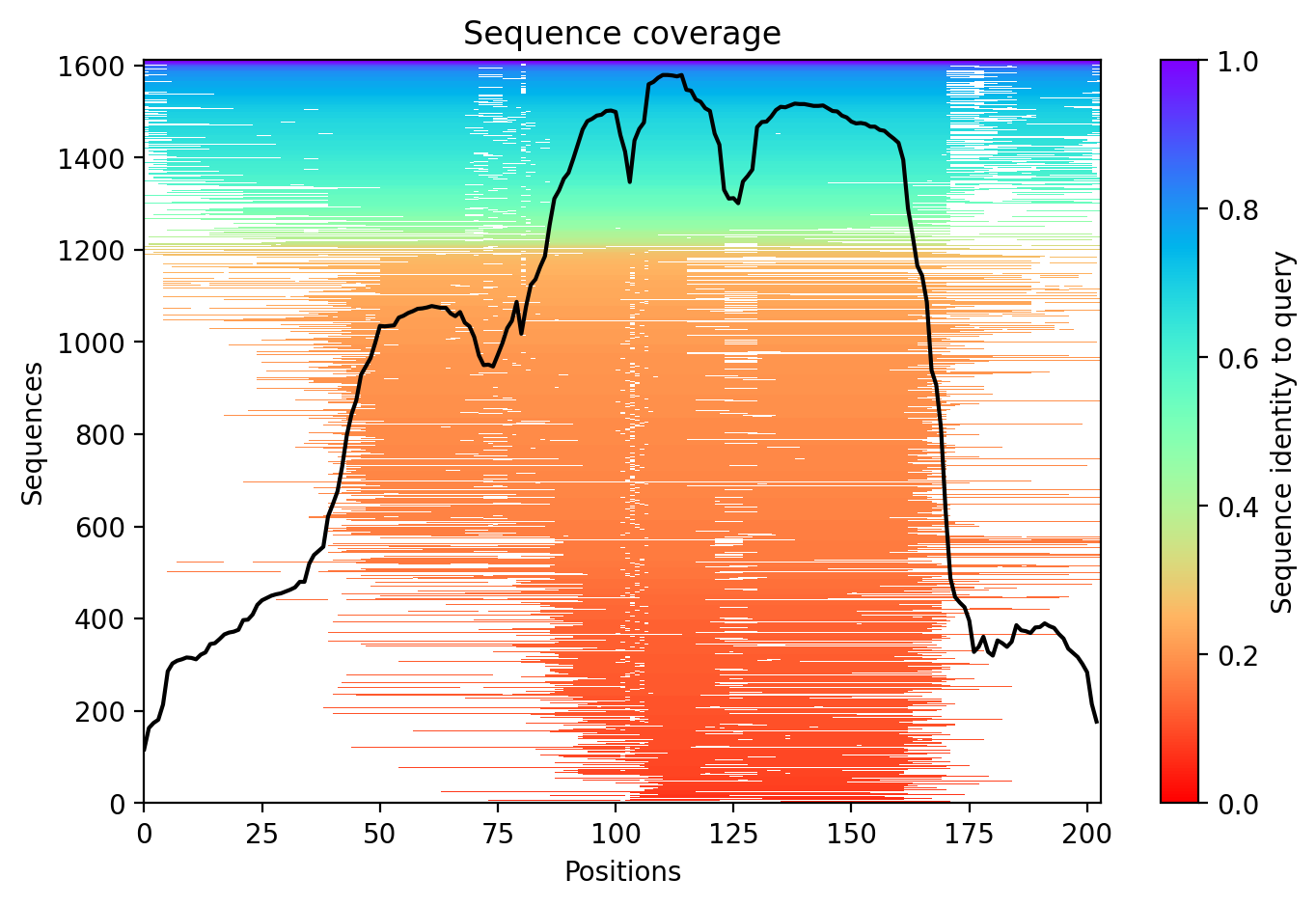

Supplement: Supplementary file 4 — Additional File 4: Dataset S4. [file 13071_2025_6771_MOESM4_ESM.zip › PhlebotomussergentiEB_01ca0/PhlebotomussergentiEB_01ca0_coverage.png]

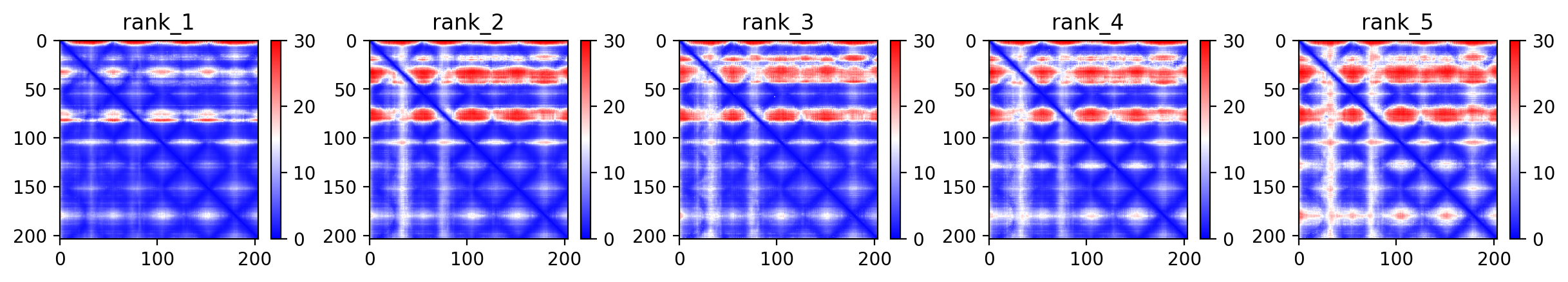

Supplement: Supplementary file 4 — Additional File 4: Dataset S4. [file 13071_2025_6771_MOESM4_ESM.zip › PhlebotomussergentiEB_01ca0/PhlebotomussergentiEB_01ca0_pae.png]

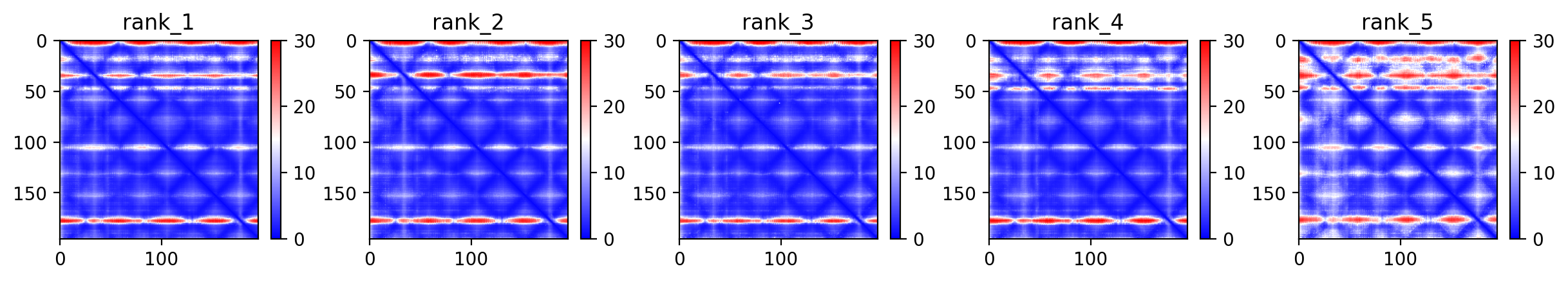

Supplement: Supplementary file 5 — Additional File 5: Dataset S5. [file 13071_2025_6771_MOESM5_ESM.zip › PhlebotomussergentiSH_ae436/PhlebotomussergentiSH_ae436_pae.png]

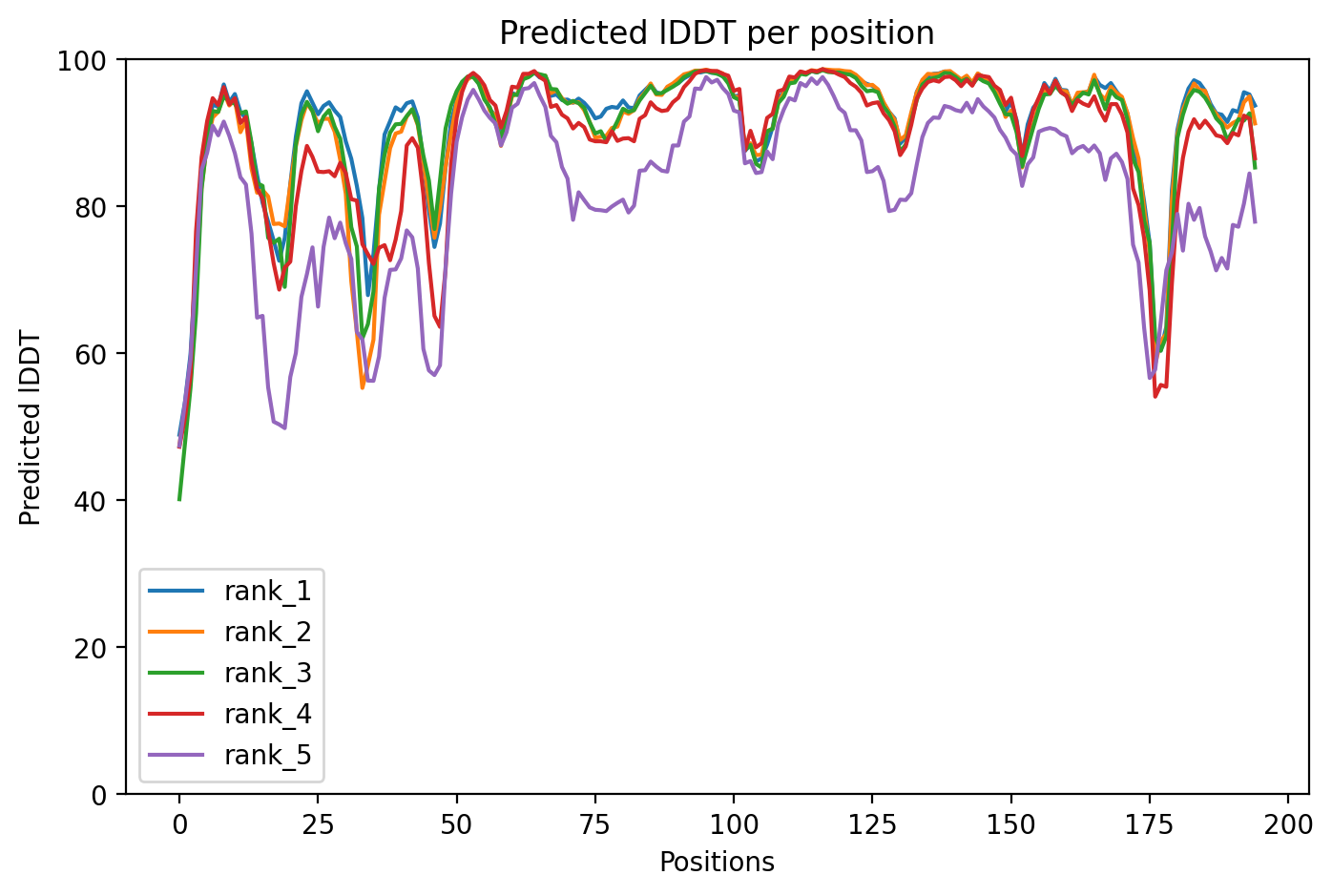

Supplement: Supplementary file 5 — Additional File 5: Dataset S5. [file 13071_2025_6771_MOESM5_ESM.zip › PhlebotomussergentiSH_ae436/PhlebotomussergentiSH_ae436_plddt.png]

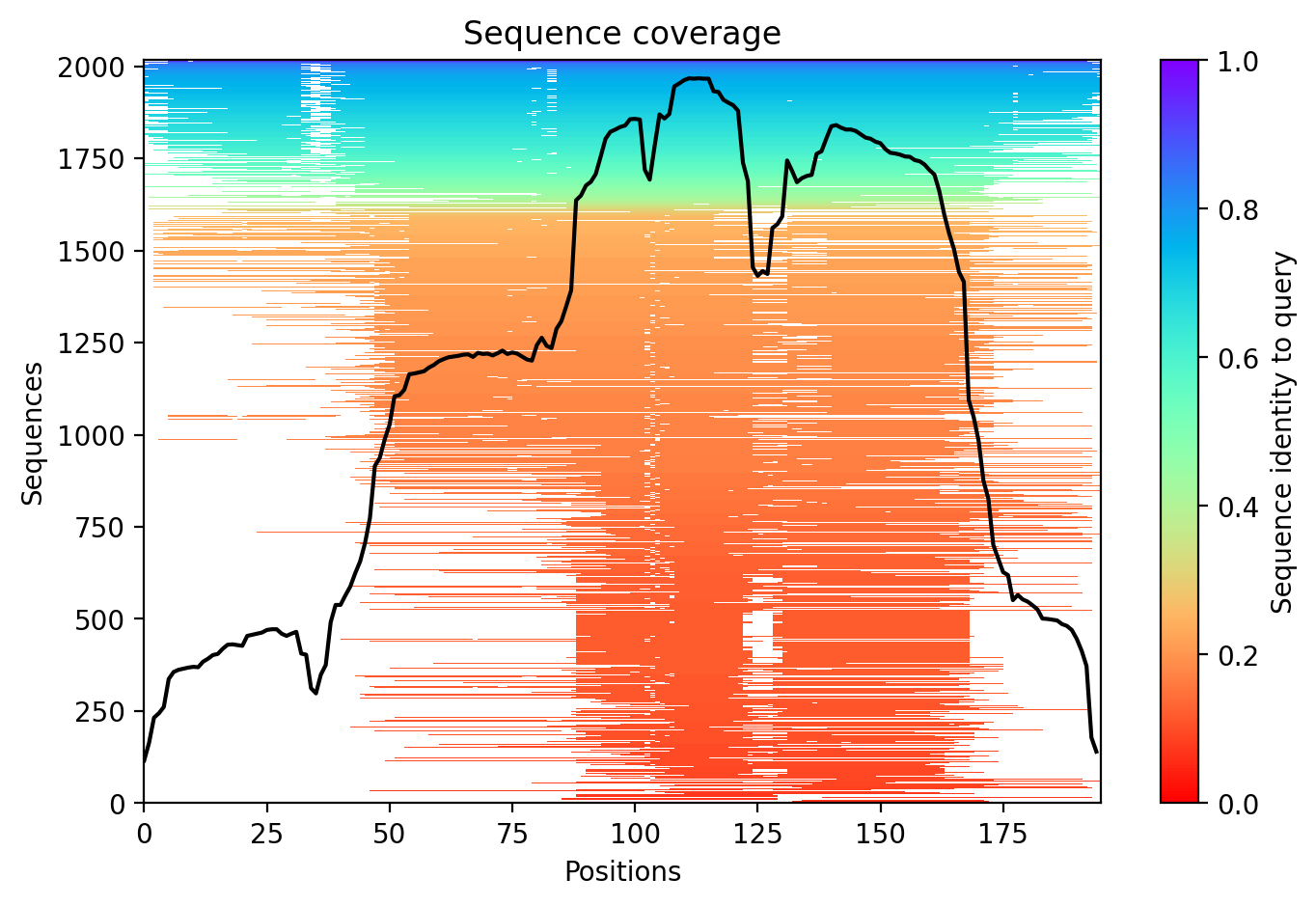

Supplement: Supplementary file 5 — Additional File 5: Dataset S5. [file 13071_2025_6771_MOESM5_ESM.zip › PhlebotomussergentiSH_ae436/PhlebotomussergentiSH_ae436_coverage.png]
